# Sildenafil Citrate Downregulates PDE5A mRNA Expression in Women with Recurrent Pregnancy Loss without Altering Angiogenic Factors—A Preliminary Study

**DOI:** 10.3390/jcm10215086

**Published:** 2021-10-29

**Authors:** Monika Kniotek, Aleksander Roszczyk, Michał Zych, Małgorzata Wrzosek, Monika Szafarowska, Radosław Zagożdżon, Małgorzata Jerzak

**Affiliations:** 1 Department of Clinical Immunology, Medical University of Warsaw, 59 Nowogrodzka St., 02-006 Warsaw, Poland; monika.kniotek@wum.edu.pl (M.K.); aleksander.roszczyk@wum.edu.pl (A.R.); michal.zych@wum.edu.pl (M.Z.); radoslaw.zagozdzon@wum.edu.pl (R.Z.); 2 Department of Biochemistry and Pharmacogenomics, Faculty of Pharmacy, Medical University of Warsaw, 1 Banacha St., 02-097 Warsaw, Poland; mmjerzak@wp.pl; 3 Laboratory of Biochemistry and Clinical Chemistry, Preclinical Research Center, Medical University of Warsaw, 1 Banacha St., 02-097 Warsaw, Poland; 4 Department of Gynecology and Oncological Gynecology, Military Institute of Medicine, 128 Szaserów St., 04-141 Warsaw, Poland; monika.szafarowska@wp.pl; 5 Department of Immunology, Transplantology and Internal Diseases, Medical University of Warsaw, 59 Nowogrodzka St., 02-006 Warsaw, Poland; 6 m-CLINIC 77/U9 Pulawska St., 02-595 Warsawa, Poland

**Keywords:** RPL, NK cells, sildenafil, PDE5A, VEGF-A, angiotensin

## Abstract

In our previous study, we showed that sildenafil citrate (SC), a selective PDE5A blocker, modulated NK cell activity in patients with recurrent pregnancy loss, which correlated with positive pregnancy outcomes. It was found that NK cells had a pivotal role in decidualization, angiogenesis, spiral artery remodeling, and the regulation of trophoblast invasion. Thus, in the current study, we determined the effects of SC on angiogenic factor expression and production, as well as idNK cell activity in the presence of nitric synthase blocker L-NMMA. Methods: NK cells (CD56^+^) were isolated from the peripheral blood of 15 patients and 15 fertile women on MACS columns and cultured in transformation media containing IL-15, TGF-β, and AZA—a methylation agent—for 7 days in hypoxia (94% N_2_, 1% O_2_, 5% CO_2_). Cultures were set up in four variants: (1) with SC, (2) without SC, (3) with NO, a synthase blocker, and (4) with SC and NO synthase blocker. NK cell activity was determined after 7 days of culturing as CD107a expression after an additional 4h of stimulation with K562 erythroleukemia cells. The expression of the *PDE5A, VEGF-A, PIGF, IL-8,* and *RENBP* genes was determined with quantitative real-time PCR (qRT-PCR) using TaqMan probes and ELISA was used to measure the concentrations of VEGF-A, PLGF, IL-8, Ang-I, Ang-II, IFN–γ proteins in culture supernatants after SC supplementation. Results: SC downregulated *PDE5A* expression and had no effect on other studied angiogenic factors. *VEGF-A* expression was increased in RPL patients compared with fertile women. Similarly, VEGF production was enhanced in RPL patients’ supernatants and SC increased the concentration of PIGF in culture supernatants. SC did not affect the expression or concentration of other studied factors, nor idNK cell activity, regardless of NO synthase blockade.

## 1. Introduction

Unexplained recurrent pregnancy loss [1] is a growing health problem worldwide. It is estimated that RPL affects over 1% of the general population and only half of the cases can be explained after a medical investigation. Each miscarriage increases the risk of another miscarriage to 15% [2,3]. Recurrent pregnancy loss [2] was defined according to the WHO definition as three or more consecutive spontaneous miscarriages before the 20th week of gestation [4,5]. ESHRE guidelines indicated immunological diagnostics in case of two idiopathic pregnancy losses [1]. Despite well-described causes of RPL, such as chromosomal abnormalities, uterine anatomical malformations, endocrine dysfunctions, thrombophilic factors, and immune disorders, the reasons remain unknown in approximately 50% of RPL cases [6]. 

It was reported that sildenafil citrate (SC) could be applied for the treatment of such complications during pregnancy as intrauterine fetal growth restriction (FGR) [7,8,9,10], low birth weight [11], preeclampsia, or idiopathic recurrent pregnancy losses [10,11,12,13,14,15]. However, recent findings have emphasized the lack of action of sildenafil on FGR and suggested further studies to assess the safety and efficacy of SC in utero, in addition to the implications on long-term health [16]. Clinical research showed that SC might increase the risk of neonatal pulmonary hypertension [17]. Nonetheless, our previous clinical research showed decreased NK activity in in vivo and in vitro research in 40 RPL patients treated with SC suppositories BEFORE conception, which correlated with positive pregnancy outcomes: live births and the lack of complications during pregnancy [12]. Therefore, SC may modulate the immune response to the trophoblast antigens and endometrial environment by the modification of NK cell function [12,13,18,19,20]. 

SC increases cellular cGMP levels through competition for the phosphodiesterase binding site with cGMP, thus inhibiting the degradation of cGMP to GMP [21]. A high level of cGMP results in increased NO production and subsequently causes the relaxation of vascular smooth muscles and increases vasodilation [7,21,22]. Vaginal sildenafil acting through NO improves uterine artery blood flow and sonographic endometrial thickness in patients with previous failed assisted reproductive cycles due to poor endometrial response. While improving uterine blood flow in the proliferative phase, NO may exert a detrimental effect on the development of the endometrium during the implantation window [11,12]. NO mediates the release of cytokines such as tumor necrosis factor-α [6,23] secreted from activated natural killer cells [24] which was implicated as a cause of implantation failure [12]. Normally, NK cells, which account for approx. A total of 70% of the decidual immune cells as so-called ‘decidual NK cells (dNK)’ [25] have a high capacity of producing cytokines such as IFN-γ, TNF-α, GM-CSF, TGF-β, and IL-10. Previous reports implied that this special population of dNK cells was involved in decidualization, angiogenesis [24], the regulation of trophoblast invasion [18], and spiral artery remodeling [26] by promoting the production of certain cytokines and factors, e.g., interferon gamma-induced protein 10 (IP-10), vascular endothelial growth factor A (VEGF-A), and IFN-γ [27]. The expression of placental growth factor (PIGF), angiogenin, endostatin, and sIL-2R increased by dNK cells may contribute to pregnancy disorders associated with poor spiral artery remodeling [25]. It was shown that angiotensin II induced vascular dysfunction dependent on IFN-γ production by NK cells [28]. Jurewicz et al. demonstrated that NK cells were fully equipped with renin–angiotensin system elements: renin, the renin receptor, angiotensinogen, and angiotensin-converting enzymes, which were capable of producing and delivering Ang II to the sites of inflammation [29]. The local renin–angiotensin system in the placenta plays an extremely important role in placental development. It was established that most of the circulating and local RAS components were over-expressed during normal pregnancy and the disruption of the balance might cause pregnancy complications [30].

dNK cells are characterized by limited cytotoxicity and they control embryo implantation and spiral artery formation [18,29]. However, several studies showed that women with recurrent pregnancy loss had a higher cytotoxic activity of NK cells both in the periphery and in the endometrium compared with healthy fertile women [31,32,33]. Our previous research showed that SC decreased peripheral blood NK activity in women with RPL treated with SC suppositories, which was correlated with positive pregnancy outcomes [12].

In 2013, Cadeira et al. proposed an in vitro model of the conversion of peripheral blood NK cells (pbNK) into induced decidual NK cells (idNK cells), which resembled dNK cells phenotypically and functionally [34]. 

In the current study we attempted to establish whether SC influenced the activity of idNK cells, obtained via the above-mentioned method, through blocking phosphodiesterase 5A (PDE5A) and increasing the level of cGMP or NO synthase activation. To determine if SC can influence idNK cell angiogenic activity, we used a real-time polymerase chain reaction to measure the mRNA levels of VEGF-A, PIGF, CXCL8 (IL-8), and PDE5A in idNK cells cultured with or without SC. Additionally, we measured VEGF-A, PLGF, IL-8, IFN-γ, angiotensin I (Ang I), and angiotensin II (Ang II) concentrations in the supernatants obtained from the cultures.

## 2. Material and Methods

The study was approved by the Bioethics Committee of the Medical University of Warsaw (No. KB/192/2015). All measurements, interventions, and blood collections were performed after informed consent had been obtained. 

### 2.1. Control Group

The control group consisted of 15 fertile women without a history of obstetric-gynecological and internal disorders. None of the subjects included in the control group reported any problems regarding conception; all subjects declared a normal course of pregnancy and delivery. Moreover, none of the control subjects were treated for any internal disorders. Women on oral hormonal contraception and other forms of hormonal treatment, or women with hormonal intrauterine devices, were excluded from the study. Transvaginal ultrasound scans were performed in all the patients between day 3 and day 5 of the menstrual cycle to reveal the normal morphology of the uterus, endometrium, and appendages.

### 2.2. Study Group

One hundred and fifty patients with RPL were evaluated. However, only fifteen patients with RPL were finally included in the study group because of very strict exclusion criteria. Recurrent pregnancy loss was defined according to the WHO definition as three or more consecutive spontaneous miscarriages before the 20th week of gestation [35]. All studied patients had experienced the last miscarriage at least 6 months before the research. Therefore, the immunological status of patients had normalized before the study. A complete medical, surgical, and social history was obtained in all cases. All the women with a history of RPL were investigated in terms of any identifiable causes of abortion. Hysterosalpingography or hysteroscopy revealed no abnormalities in the patients’ uteri. The study group underwent peripheral blood chromosome assessment which revealed normal karyotypes. Patients with anatomic, genetic, microbiological, immunological, and hormonal causes of abortions were excluded from the research. The women with RPL were tested for thrombophilia and immunological markers such as aPL and none of them exhibited any defects. The characteristics of the study group, including age and the number of spontaneous pregnancy losses, are listed in Table S1. 

## 3. Methods

### 3.1. The Isolation of Peripheral Blood Mononuclear Cells and CD56^+^ Cells

Peripheral blood mononuclear cells (PBMC) were isolated from the peripheral blood of 15 women with recurrent miscarriages and 15 healthy volunteers via Ficoll gradient centrifugation. After being washed twice in phosphate-buffered saline (PBS, Biomed, Lublin, Poland), the cells were suspended in 1 mL of cold MACS buffer (MiltenyiBiotec, Auburn, CA, USA) The cells were counted and stained according to the manufacturer’s instructions with the appropriate amount of CD56^+^ microbeads. After washing, the stained cells were separated with MidiMACS manual separator (MiltenyiBiotec, Auburn, CA, USA) according to the manufacturer’s instructions (MiltenyiBiotec, Auburn, CA, USA). After isolation, we obtained approximately 2 × 10^6^ CD56-positive cells with 96% purity.

### 3.2. Cell Culture

Isolated CD56-positive cells were cultured in 24-well plates (SPL Life Sciences Co., Ltd., Naechon-Myeon, Gyeonggi-do, South Korea), in Opti-MEM Reduced Serum Media (Gibco, Thermo Fisher Scientific, Waltham, MA, USA) containing 10% FCS (MERK, KGaA, Darmstadt, Germany), 1 U/ML penicillin/streptomycin/100 μg/mL [11], 2 mM glutamine (Sigma Aldrich), 1 mM sodium pyruvate (Fluka), nonessential amino acids ((MERK, KGaA, Darmstadt, Germany), 55 mM 2-mercapthoethanol, 10 ng/mL recombinant human IL-15 (MERK, KGaA, Darmstadt, Germany), 2 ng/mL recombinant human TGF-β-1 (R&D), and 1 μM 5-aza-2′deoxycytidine (AZA, (MERK, KGaA, Darmstadt, Germany), in a hypoxic (94% N_2_, 5% CO_2_, 1% O_2_) environment [24]. 

The cells were cultured at a concentration of 1 × 10^6^/mL in 4 variants: NK cells in the medium, NK cells with 400 ng/mL of sildenafil citrate (MERK, KGaA, Darmstadt, Germany), NK cells with 500 μM of NOS inhibitor—NG-Monomethyl-L-arginine, monoacetate salt (L-NMMA, (MERK, KGaA, Darmstadt, Germany), NK cells with 500 μM of L-NMMA and SC (both from MERK, KGaA, Darmstadt, Germany), [36,37]. The concentration of SC used in the experiments was consistent with the physiological concentration of 200 mg of orally administered Viagra in the blood of a healthy man [38]. Hypoxic conditions were obtained by culturing cells in a sealed anaerobic workstation incubator (Modular Incubator Chamber, MIC-101, Billups-Rothenberg, Inc., Del Mar, CA, USA), incorporated with a gas flow measurement system (DFM 3002, Biogenet, Józefów, Poland) and flushed with a mixture of 1% O_2_, 5% CO_2_, and 94% N_2_. The entry and exit ports of the chambers were subsequently clamped, and the chambers were placed in a 37 °C incubator. After 5 days of culturing, the cells were harvested for the determination of idNK cell degranulation and activity or gene expression pattern. 

### 3.3. Degranulation of idNK Cells—CD107a Expression Determination

After 5 days of culturing, idNK cells were seeded with the E:T ratio of 2:1 and 1:1 in falcon tubes in RPMI full medium and incubated for 4 h at 37 °C and 5% CO_2_. For functional assays, anti-CD107a APC was added together with monensin (GolgiStop TM, Becton Dickinson, Franklin Lakes, NJ, USA) and brefeldin A (GolgiPlug TM, Becton Dickinson, Franklin Lakes, NJ, USA) at the beginning of the assay. At the end of the assay, the cells were stained for CD3-FITC, CD56-PE surface markers to identify the CD3^-^CD56^+^ idNK-cell subsets. Gating strategy and cut-off values of positive fluorescence were based on a fluorescence minus one (FMO) experiment and are shown in supplementary data (Figure S1). Cell readouts were acquired using a Becton Dickinson FACSCanto II cytometer (BD FACS Canto II, Becton Dickinson, Franklin Lakes, NJ, USA) and analyzed with BD FACS Diva 6.1.3. software. Analyses were conducted on live cells only [39].

### 3.4. The Determination of the Gene Expression of Selected Angiogenic Factors

Cultures for gene expression determination were performed in 2 versions: The culture of idNK cells and idNK cells supplemented with SC. The cells were centrifuged at 800 *g* for 10 min., culture supernatants were collected and frozen at −80 °C for subsequent analysis with the enzyme-linked immunosorbent assay (ELISA) and idNK cells were suspended in a lysis buffer (RLT buffer, Qiagen, Hilden, Germany). RNA was extracted from frozen idNK cells using the guanidinium-thiocyanate-based RNA extraction (Qiagen RNeasy Mini Kit, Qiagen, Valencia, CA) followed by column-based purification. RNA concentration and the purity were evaluated with a micro-volume UV-Vis spectrophotometer (Quawell Q3000, Quawell Technology Inc., San Jose, CA, USA). Quantitative RT-PCR was carried out with the RNA-to-C_T_™ 1-Step kit [(Applied Biosystems (AB), Foster City, CA, USA]. The TaqMan Gene Expression Assays (Thermo Fisher Scientific, Inc., Waltham, MA, USA) were performed with the use of a ViiA™7 Real-Time PCR system (Applied Biosystems; Thermo Fisher Scientific, Inc.) under the following thermocycling conditions: 48 °C for 15 min, 95 °C for 10 min and 40 cycles of 95 °C for 15 sec and 60 °C for 1 min. TaqMan Probes used for RT-PCR are presented in Table 1. Data were normalized to the reference genes (GAPDH and B2M, Table 1) and the relative expression level of each target gene was expressed as 2^−(Ct; Target gene −Ct; Reference gene)^. All qRT-PCR experiments were run in triplicate, and the mean value was used for the determination of mRNA levels [40,41]. 

### 3.5. The Determination of the Concentration of Angiogenic Factors

The concentrations of selected cytokines and angiogenic factors (VEGF-A, PIGF, IL-8, IFN-γ, Ang I, Ang II) were measured in idNK culture supernatants twice via the enzyme-linked immunosorbent assay (ELISA) according to the manufacturer’s instructions. The concentrations of cytokines were calculated from a standard curve of linear regression according to the manufacturer’s instructions (ELISA kits, Bioassay Technology Laboratory, Shanghai, China). The sensitivity of ELISA kits were: VEGF-A—1.52 ng/L, PIGF—4.02 ng/mL, INF-γ—0.49 ng/mL, IL-8—2.51 ng/L, angiotensin I (Ang I) < 75 pg/mL, angiotensin II (Ang II)—18.75 pg/mL. The intra-assay CV was < 8% and the inter-assay CV was < 10% for all used kits [42].

### 3.6. Statistical Analysis 

All statistical analyses were performed with Graph Pad Prism 9.00. The normal distribution of data was determined with the Shapiro–Wilk test. In order to determine the statistical significance between the control and study group samples, the unpaired *t*-test was used in case of the normal distribution of data, and the Mann–Whitney U test was used in case of non-normal distribution. The analyses of data inside the groups (samples after culturing with SC) were performed with the Wilcoxon signed-rank test in case of non-normal distribution and the paired *t*-test for the normal distribution of samples. The *p* values below 0.05 (*p* < 0.05) were considered statistically significant. The data were shown as the median and interquartile range (IQR) in the figures.

## 4. Results

No difference was observed as regards the age between RPL patients (36.70 ± 4.48) and healthy women (37.40 ± 1.90). 

### 4.1. The Determination of the Gene Expression of PDE5A and Selected Angiogenic Factors

**Figure 1:**
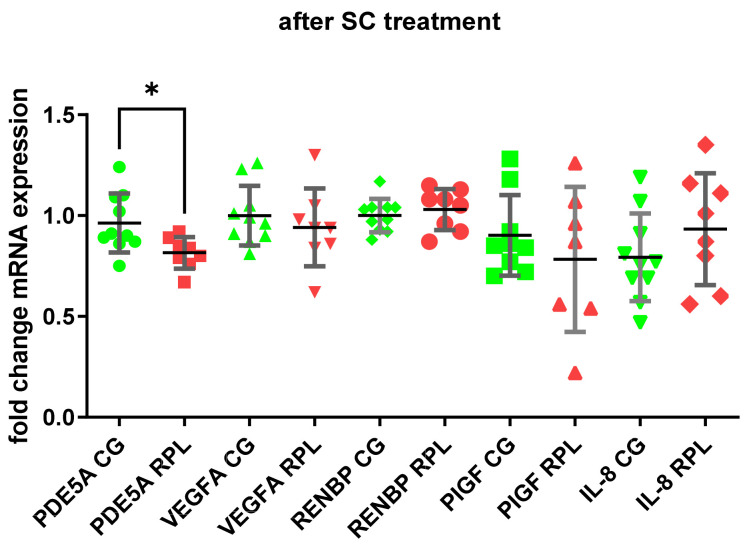
*PDE5A*, *VEGF-A, ANG-I, PIGF, IL-8* expression after SC treatment in the idNK cells of healthy and RPL women, data shown as the mean ± SD compared with idNK cells maintained without SC (green points, CG—control group, *n* = 10; red points—RPL patients, *n* = 8; statistical significance * *p* < 0.05).

**Figure 2:**
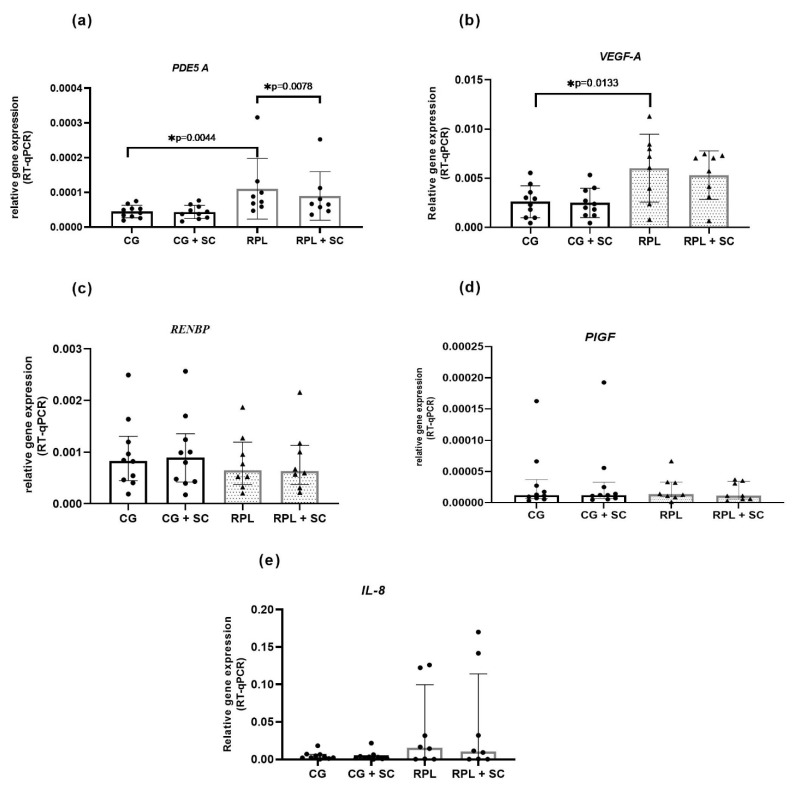
The expression of *PDE5A* and *VEGF-A* is upregulated in the idNK cells of patients with RPL. SC downregulated *PDE5A* gene expression in RPL group, (**a**) a genomic meta-analysis of PDE5A gene expression in idNK cells, (**b**) a genomic meta-analysis of *VEGF-A* gene expression in idNK cells (**c**) a genomic meta-analysis of *RNEBP* gene expression in idNK cells, (**d**) a genomic meta-analysis of *PIGF* gene expression in idNK cells, (**e**) a genomic meta-analysis of *IL-8* gene expression in idNK cells; data are shown as the median and interquartile range, CG—control group *n* = 10, RPL patients *n* = 8, circles and triangles represents single samples; SC—sildenafil citrate 400 ng/mL, statistical significance marked as star—* *p* < 0.05.

The PDE5A enzyme was expressed in idNK cells, and its expression was significantly higher in RPL women than in healthy women (Figure 1 and Figure 2) The addition of 400 ng/mL SC to the culture decreased the expression of *PDE5A* in RPL patients (Figure 1). We also found a significantly higher expression of *VEGF-A* in RPL patients than in the control group (Figure 2). No significant differences occurred in the mRNA expression of *PIGF, RNEBP,* and *CXCL8 (IL-8)* between the studied groups. However, the expression of IL-8 showed a wide range in RPL and control group (Figure 1 and Figure 2).

### 4.2. The Levels of Angiogenic Factors in the Culture Supernatants of idNK Cells

**Figure 3:**
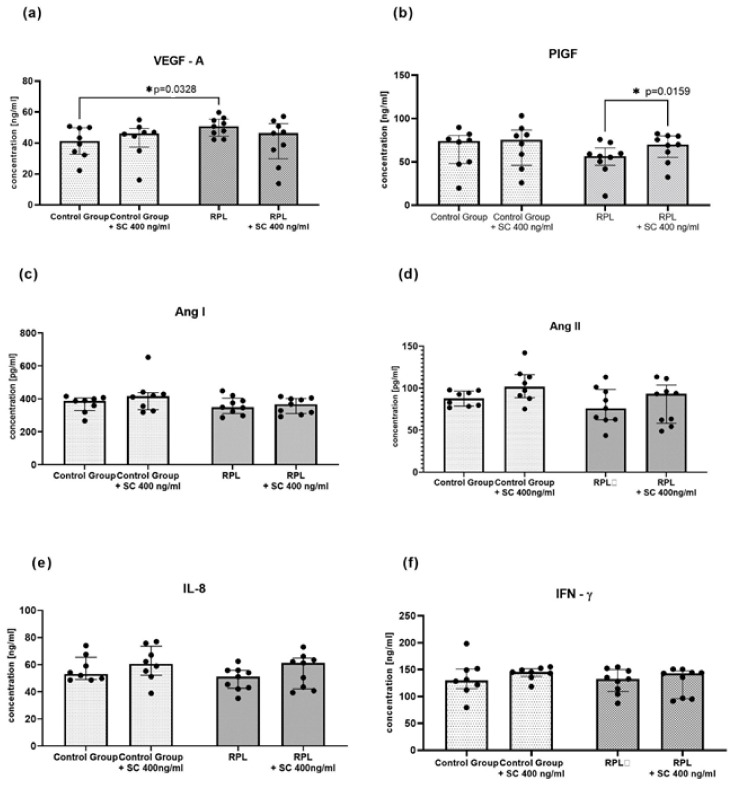
The concentrations of angiogenic factors and cytokines in idNK cultures with and without sildenafil citrate: (**a**) VEGF-A, (**b**) PIGF, (**c**) Ang I, (**d**) Ang II, (**e**) IL-8; (**f**) IFN-γ; Control group *n* = 9, RPL patients *n* = 8; dots on the figures reflects numbers of determined samples, SC—sildenafil citrate 400 ng/mL, statistical significance marked as star—* *p* < 0.05.

The concentrations of IL-8, IFN-γ, and angiogenic factors (VEGF-A, PIGF, angiotensin I, angiotensin II) were measured in the culture supernatants of idNK cells with the ELISA method. Surprisingly, we found no difference in protein levels between the studied groups before or after SC treatment (Figure 3). Similarly to gene expression, we found that VEGF-A concentration was upregulated in RPL patients. Moreover, PIGF level was enhanced after SC treatment. 

### 4.3. idNK Cell Activity and CD107a Expression

The flow cytometry determination of idNK cell cytotoxicity and degranulation did not reveal any differences between the groups. The blockage of NO synthase with L-NMMA did not alter idNK activity or the expression of CD107a in the studied groups. Sildenafil citrate did not influence idNK cell function in any of the studied variants (Figure S2). 

## 5. Discussion

Decidualization, which involves a dramatic morphological and functional differentiation of human endometrial stromal and immune cells, plays an important role in promoting placental formation to support pregnancy [43]. dNK cells are the most abundant population of immune cells in the decidua. Almost a decade has passed since researchers pointed out that increased pbNk cell activity reflected the activity of decidual NK cells [29]. Further studies on dNK cells revealed their regulatory function during a physiological pregnancy, including the creation of optimal conditions for blastocyst invasion, control of the invasion process depending on gestation terms, as well as taking part in the uterus spiral artery remodeling and uterus–placenta normal blood flow establishment [25,44,45,46]. 

In our previous study, we observed that sildenafil citrate decreased pbNK cell activity, and improved uterine blood flow and the endometrial thickness of women with RPL [12]. Thus, in the present study, we verified the influence of SC on the activity and angiogenic properties of idNK cells. We found that supplementing the culture with a low dose of sildenafil citrate downregulated *PDE5* gene expression. To the best of our knowledge, it is the first study investigating the expression of *PDE5A* phosphodiesterase in human NK cells and idNK cells. Furthermore, we showed the expression of the gene was significantly higher in RPL patients, which suggests a faster conversion of cGMP to GMP in the dNK cells of RPL patients. CGMP indirectly activates NO synthases, and it is known that nitric oxide (NO) improves endometrial thickness by enhancing uterine blood flow (7, 9). It also improves NK activity [36]. The shortage of cGMP in the cells of RPL patients may disturb NO release and, subsequently, cause an inadequate cytotrophoblast invasion of the uterine spiral arteries and endometrium growth, as well as embryonic cell proliferation decrease [12,47,48,49]. The downregulation of *PDE5* gene expression by SC may restore the required level of cGMP and improve NO production demanded during trophoblast implantation. However, further research is needed.

We performed cultures of idNK cells with and without a blocker of NO synthase (L-NMMA) supplementation in a hypoxic (1% of oxygen) environment to determine if SC influenced iNOs synthase and modulated NK activity. Cifoni et al. showed that NO synthase inhibitors impaired NK cell-mediated target cell killing [50]. However, L-NMMA, SC, or both compounds did not affect idNK activity towards K562 tumor cells or the expression of lysosomal-associated membrane protein-1 (LAMP-1; CD107a) in our research. Sanson and Malagoni reported that even in the presence of L-NMMA, NO generation occurred following hypoxia [51]. Therefore, it was probably hypoxia which abolished the effect of L-NMMA in our study. Moreover, transformation media for idNK cell cultures contained IL-15 cytokine which plays a dominant role in NK cell activation. It was shown that brief priming with IL-15 markedly enhanced the antitumor response of CD56^bright^ NK cells [52]. Furthermore, we observed no changes in the CD107a expression on idNK cells after SC addition. The data are in opposition to our previous results obtained from the cultures of PBMC, where SC decreased pbNK cell activity after 48 h of culturing [12]. It may also suggest an influence of other immune cells controlling NK activity and the impact of hypoxia on idNK cells. According to some authors, hypoxia improves NO release, and subsequently improves NK cell activity [6,24,50].

It was found that hypoxia was the principal regulator of VEGF expression [53]. We noted a higher expression of VEGF mRNA in the idNK cells of RPL patients in hypoxia which was reflected in the concentration of VEGF in culture supernatants. Atalay et al. and Pan et al. reported a higher level of VEGF in the serum of RPL patients than in fertile women [54,55]. It was demonstrated that VEGF increased microvascular permeability and promoted coagulation, which is directly related to the pathogenesis of preeclampsia [23]. Others claimed that VEGF-A level in the serum of RPL patients was not correlated with NK cell function and level [56].

Several studies demonstrated that dNK cells produced angiogenic factors and cytokines such as PIGF, IL-8, IFN-γ, Ang I, Ang II, which influenced endothelial integrity, and the imbalanced expression resulted in endothelial barrier dysfunction and vascular permeability in RPL patients [18,27,57]. In 2016, Cavalli et al. showed that idNK cells obtained from pbNK cells by culturing in transformation media in hypoxia expressed similar factors [24]. After the implementation of cultures similar to those described by Cavali et al., we tested the levels of the aforementioned factors in culture supernatants. Turner et al. reported that women who required emergency operative deliveries as a result of fetal distress had low pre-labor levels of placental growth factor (PIGF), which declined as labor progressed. During a recent phase 2 randomized controlled trial (RCT), they showed that women in the placebo cohort had a greater decline in PIGF levels than those receiving SC [58]. A clinical case study of a 26-year-old woman with severe preeclampsia showed that SC increased the PLGF level measured in the serum [59]. Our RPL group of patients had lower concentrations of PIGF than the fertile group of women, which was not reflected in gene expression and SC improved the concentration of PLGF as in the mentioned studies. However, we did not test RPL women during labor or pregnancy. 

It was widely reported that SC influenced angiogenic factor production. There is evidence that sildenafil decreased IL-8 level in the serum, as well as gene expression in the PBMC cultures of patients with diabetic cardiomyopathy [60]. Other researchers showed that SC decreased the production of IL-6, IL-8 and ROS in dermal fibroblast cultures isolated from patients with systemic sclerosis [60]. Dias et al. confirmed that sildenafil attenuated the morphofunctional deleterious effects of Ang II on resistance vessels [61]. Chiu et al. found that SC improved renin secretion in the serum of men [62]. However, the present research revealed no such effect of SC in our in vitro model of idNK cells. It was also reported that sildenafil reduced ionized calcium-binding adapter molecule 1 (Iba-1), IFN-γ, and IL-1β levels in an inflammatory demyelination model in INOS knockout mice [61,63]. We did not notice such a phenomenon concerning idNK cell cultures in the current research as well as in our previous research, SC did not influence the percentage of T lymphocytes producing IFN–γ in the PBMC cultures in healthy men [64].

We are aware that the presented data are preliminary, but they may aid in further understanding of SC effect on idNK cells and the practical use of idNK cells in the studies of the pathology of idiopathic recurrent pregnancy loss.

## Figures and Tables

**Table 1:** TaqMan probes used for RT-PCR in determination of the gene expression of selected angiogenic factors.

Gene Name	Gene Symbol	Assay ID
Phosphodiesterase 5A	PDE5A	Hs00153649_m1
Vascular endothelial growth factor A	VEGF-A	Hs00900055_m1
Placental growth factor	PIGF	Hs00182176_m1
Renin binding protein	RENBP	Hs00234138_m1
C-X-C motif chemokine ligand 8	CXCL8 (IL-8)	Hs00174103_m1
Glyceraldehyde-3-phosphate dehydrogenase	GAPDH	Hs99999905_m1
Beta-2-microglobulin	B2M	Hs99999907_m1

## Data Availability

The data presented in this study are available on request from the first author.

## Supplementary Materials

The following are available online at https://www.mdpi.com/article/10.3390/jcm10215086/s1, Table S1: Characteristics of RPL patients included to the study group; Figure S1: Gating strategy for CD107a expressing idNK cells (CD56+ CD3-) and NKT cells (CD56+ CD3+); Figure S2: The effect of sildenafil on the degranulation (CD107a expression) of idNK cells determined after 5 days of culturing in transformation media and hypoxia, results are showed as the median and IQR, CG—control group, RPL—study group, 500μM L-arginine iNOS inhibitor (L-NMMA), SC—sildenafil citrate 400 ng/ml).

## Abbreviations

Ang II	Angiotensin II
AZA	5-aza-2′deoxycytidine
cGMP	cyclic guanosine monophosphate
dNK	decidual NK cells
GMP	guanosine monophosphate
idNK	induced decidual NK cells
IL	interleukin
IUR	intrauterine growth restriction
L-MNNA	NG-Monomethyl-L-arginine
NO	nitric oxide
NOS	nitric oxide synthase
PBS	phosphate-buffered saline
PDE-Is	phosphodiesterase inhibitors
PDEs	phosphodiesterases
RAS	renin–angiotensin system
RPL	recurrent pregnancy loss
RSA	recurrent spontaneous abortions
SC	sildenafil citrate
