# Supplementary material for: Sildenafil Citrate Downregulates PDE5A mRNA Expression in Women with Recurrent Pregnancy Loss without Altering Angiogenic Factors—A Preliminary Study
Source: J Clin Med. 2021 Oct 29;10(21):5086. doi: 10.3390/jcm10215086 (PMC8584603; doi:10.3390/jcm10215086)
Supplement: Supplementary file 1 [file jcm-10-05086-s001.zip › jcm-1336953-supplementary.pdf]

Sildenafil Citrate Downregulates PDE5A mRNA Expression in Women with Recurrent Pregnancy Loss without Altering Angiogenic Factors—A Preliminary Study

Monika Kniotek[1], Aleksander Roszczyk[1], Michał Zych[1], Małgorzata Wrzosek[3], Monika Szafarowska[2], Radosław Zagożdżon[1,4], Małgorzata Jerzak[2,5]

[1]Department of Clinical Immunology, Medical University of Warsaw, 59 Nowogrodzka St., 02-006 Warsaw, Mazovieckie voivodeship, Poland

[2]Department of Gynecology and Gynecologic Oncology, Military Institute of Health and Sciences, 128 Szaserów St., 04-141 Warsaw, Mazovieckie voivodeship, Poland

[3]Department of Biochemistry and Pharmacogenomics, Faculty of Pharmacy and Laboratory of Biochemistry and Clinical Chemistry at the Preclinical Research Center, Medical University of Warsaw, 1 Banacha St., 02-097 Warsaw, Poland

[4]Department of Immunology, Transplantology, and Internal Diseases, Medical University of Warsaw, 59 Nowogrodzka St., 02-006 Warsaw, Mazovieckie voivodeship, Poland

[5] m-CLINIC 77 / U9 Pulawska St., 02-595 Warsawa, Poland

Corresponding author: Małgorzata Wrzosek, PhD, ORCID no: 0000-0002-9820-3871

Department of Biochemistry and Pharmacogenomics, Division of Biochemistry and Clinical Chemistry, Medical University of Warsaw

1 Banacha St., 02-097 Warsaw, Poland

Correspondence: malgorzata.wrzosek@wum.edu.pl

Phone number: +48 22 572 07 35

Characetristics	Value (range)
Age (years), mean (range)	36 (33-42)
Number of pregnancies – mean (range)	4.5 (2-7)
• number of miscarriages – mean (range)	3.09 (2-5)
Vitamin D (ng/ml)	32 (16.6-52.8)
AMH (ng/ml)	5.5 (1.9-12.4)
Homocysteine (mcmol/L)	9.38 (6.2-14.94)
Hashimoto's disease (number of patients)	n=1
Insulin resistance (number of patients)	n=3
Antiphospholipid syndrome (number of patients)	n=0
Thrombophilia (number of patients with confirmed thrombophilia)	
• Factor V Leiden pathogenic variant	n=0
• Prothrombin G20210A pathogenic variant	n=0
• Antithrombin deficiency (activity <60%)	n=0
• Protein S deficiency (functional assay <55%)	n=0
• Protein C deficiency (activity <65%)	n=0
• Antiphospholipid antibodies	n=0
MTHFR gene polymorphisms (number of patients with confirmed polymorphisms)	
• A1298C	n=4 (heterozygous)
• C677T	n=4 (heterozygous)
PAI-1 Gene Polymorphism (number of patients with confirmed polymorphisms)	
• 4G/5G	n=4 (heterozygous)

Table S1. Characteristics of RPL patients included to the study group.

a)

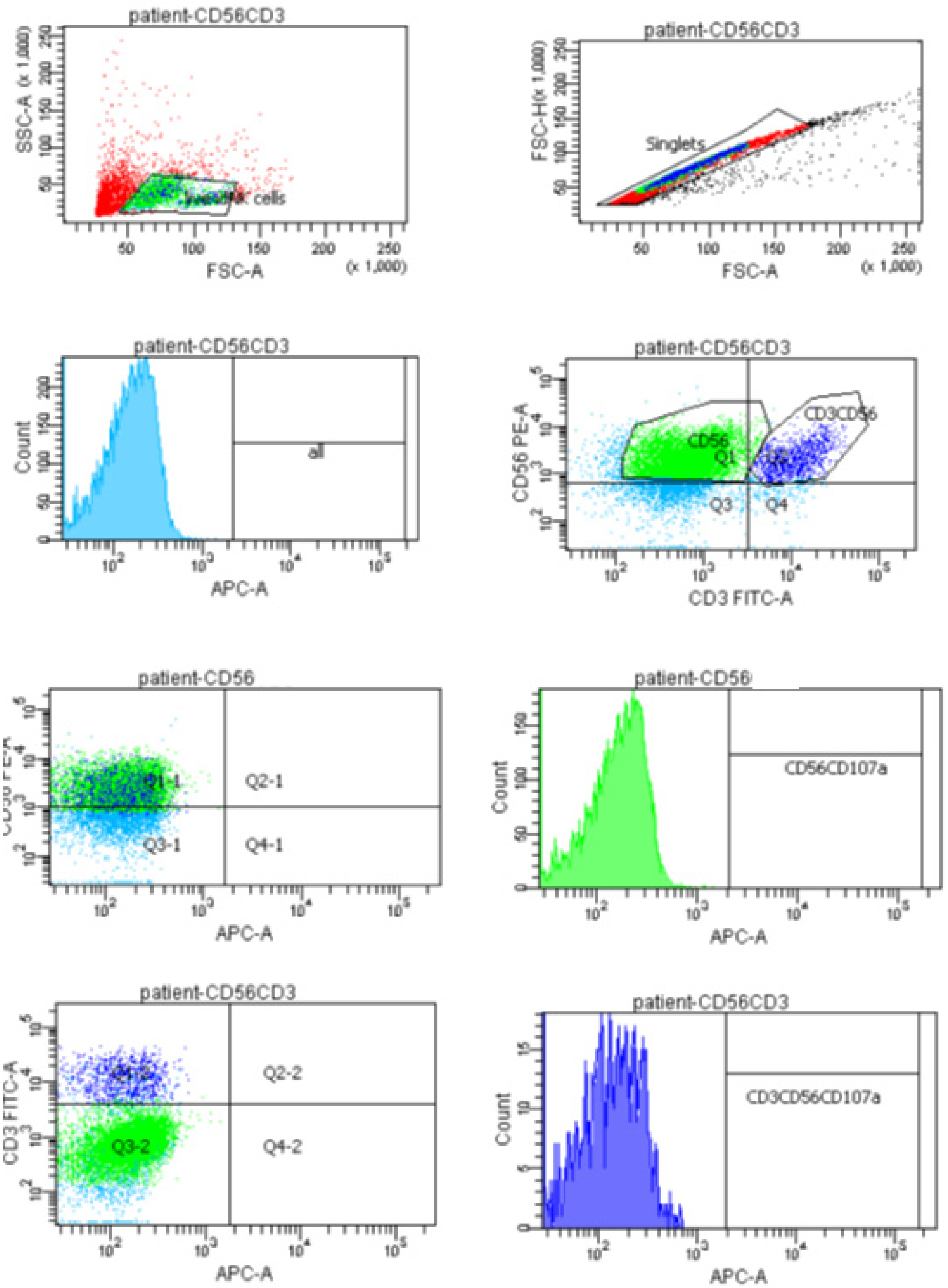
patient-CD56CD3
SSC-A (x 1,000)
FSC-A
(x 1,000)
cells
patient-CD56CD3
FSC-H(x 1,000)
Singlets
FSC-A
(x 1,000)
patient-CD56CD3
Count
all
APC-A
patient-CD56CD3
CD56 PE-A
CD3CD56
CD56
Q1
Q3
Q4
CD3 FITC-A
patient-CD56
Q2-1
Q3-1
Q4-1
APC-A
patient-CD56
Count
CD56CD107a
APC-A
patient-CD56CD3
CD3 FITC-A
Q2-2
Q3-2
Q4-2
APC-A
patient-CD56CD3
Count
CD3CD56CD107a
APC-A

b)

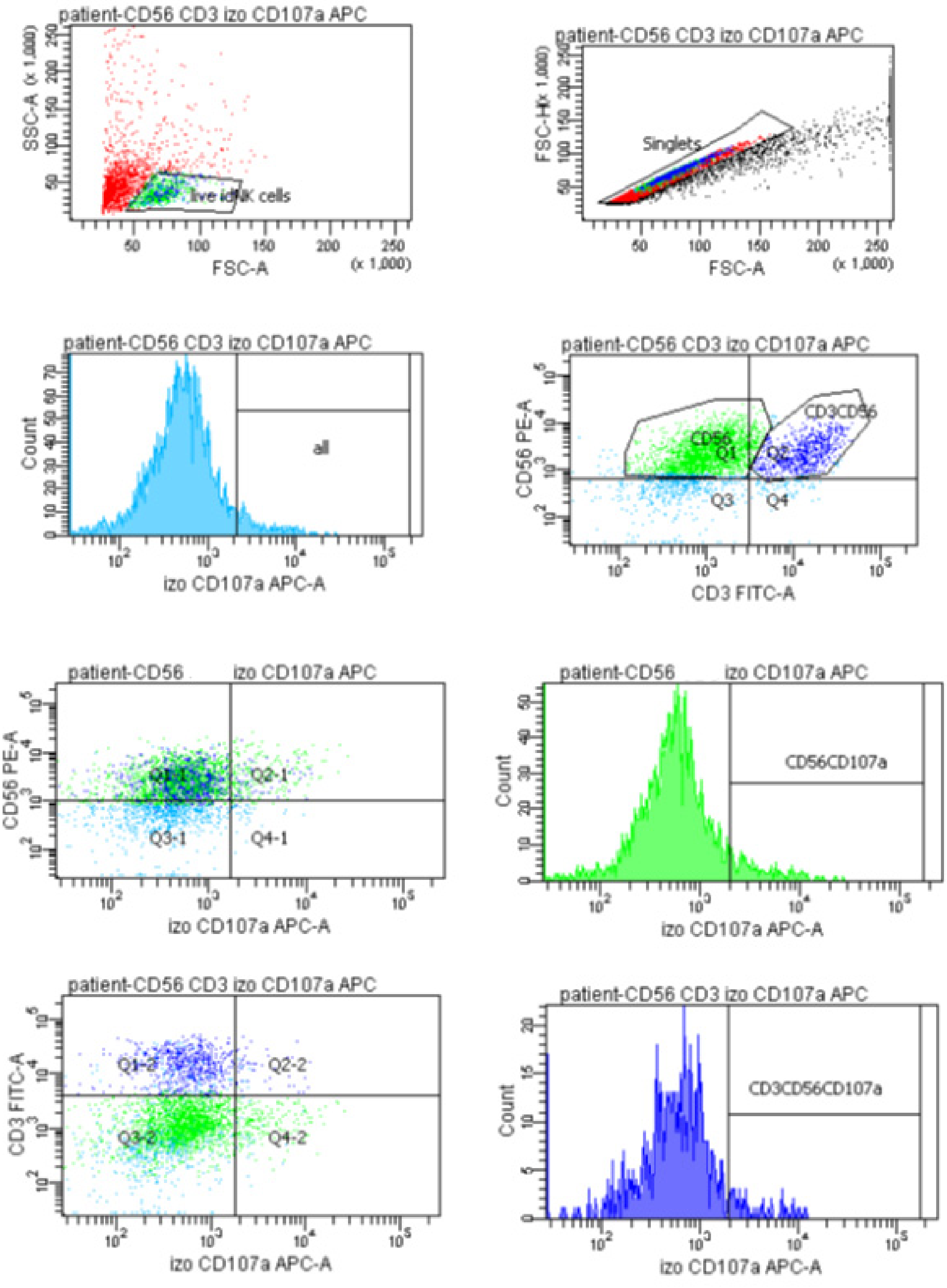
patient-CD56 CD3 izo CD107a APC
SSC-A (x 1,000)
FSC-A
(x 1,000)
Singlets
FSC-H
Count
izo CD107a APC-A
all
CD56 PE-A
CD3 FITC-A
CD56
CD3CD56
Q1
Q2
Q3
Q4
patient-CD56
izo CD107a APC
Q1-1
Q2-1
Q3-1
Q4-1
CD56CD107a
Q1-2
Q2-2
Q3-2
Q4-2
CD3CD56CD107a

c)

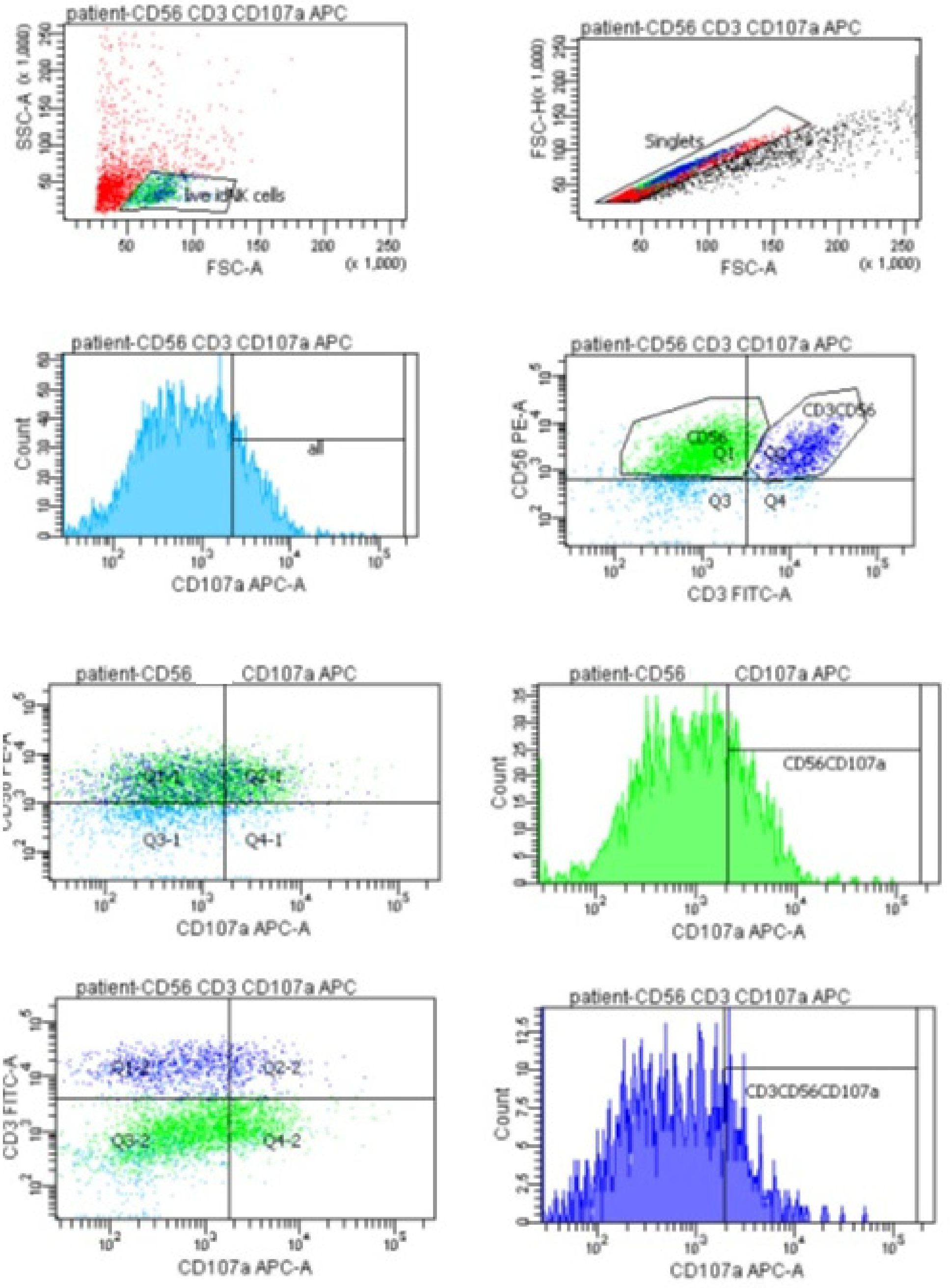
patient-CD56 CD3 CD107a APC
SSC-A (x 1,000)
live idNK cells
FSC-A
(x 1,000)
patient-CD56 CD3 CD107a APC
FSC-H (x 1,000)
Singlets
FSC-A
(x 1,000)
patient-CD56 CD3 CD107a APC
Count
all
CD107a APC-A
patient-CD56 CD3 CD107a APC
CD56 PE-A
CD56
CD3CD56
Q1
Q2
Q3
Q4
CD3 FITC-A
patient-CD56
CD107a APC
Q1-1
Q2-1
Q3-1
Q4-1
CD107a APC-A
patient-CD56
CD107a APC
Count
CD56CD107a
CD107a APC-A
patient-CD56 CD3 CD107a APC
CD3 FITC-A
Q1-2
Q2-2
Q3-2
Q4-2
CD107a APC-A
patient-CD56 CD3 CD107a APC
Count
CD3CD56CD107a
CD107a APC-A

Figure S1. Gating strategy for CD107a expressing idNK cells ($CD56^+ CD3^-$) and NKT cells ($CD56^+ CD3^+$), a) CD3CD56 and CD56-positive cell gating, singlet distributions were determined on the FSC-H/FSC-A axis, lymphocytes were identified as 50-150 x 10^3 cells on FSC, CD56-positive cells were identified among lymphocytes with red PE – fluorescence above 10^3 on Y axis on a logarithmic scale, CD3CD56-positive cells were identified with $8x10^2$ red PE-fluorescence on Y axis and $2x10^3$ green FITC-fluorescence on X axis c) FMO control for CD107a-APC expressed on CD3-FITC and CD56-PE-positive cells after K562 stimulation, APC-negative cells were identified with APC fluorescence below $1x10^3$ on a dot plot as well as on histograms for CD56 and for CD3CD56-positive cells, d) CD107a expression after activation with K562 cells on CD3 and CD56-positive cells, CD56-positive cells were identified with red PE-fluorescence above 10^3 on Y axis on a logarithmic scale, CD3CD56-positive cells were identified with $8x10^2$ red PE-fluorescence on Y axis and $2x10^3$ green FITC-fluorescence on X axis, CD56CD107a and CD3CD56CD107a APC-positive cells were identified with APC fluorescence above $1x10^3$ on X axis on dot plots for CD56 and CD3CD56 cells as well as on histograms.

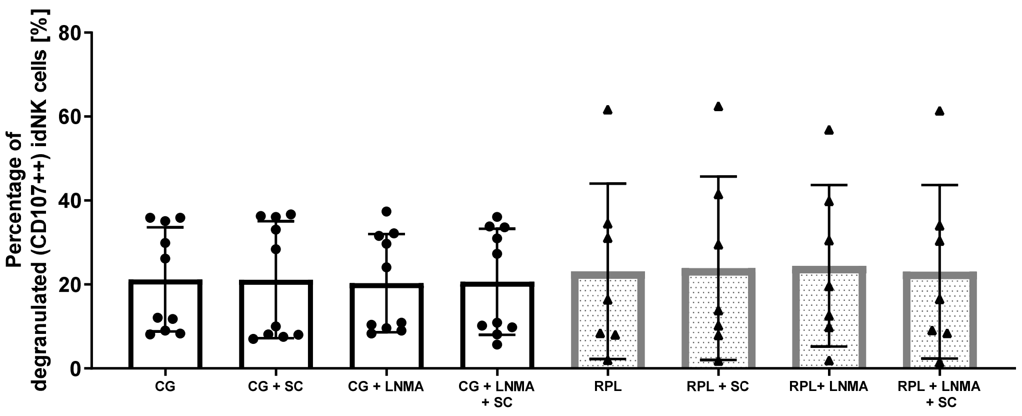

Figure S2. The effect of sildenafil on the degranulation (CD107a expression) of idNK cells determined after 5 days of culturing in transformation media and hypoxia, results are showed as the median and IQR, CG – control group, RPL – study group, 500μM L-arginine iNOS inhibitor (L-NMMA), SC – sildenafil citrate 400 ng/ml).
